# Supplementary material for: New insights on the Feeding Site and Salivation by Lutzomyia longipalpis (Diptera: Psychodidae) During Blood Ingestion on Host Skin
Source: PLoS Negl Trop Dis. 2026 Aug 24;20(8):e0014067. doi: 10.1371/journal.pntd.0014067 (PMC13568499; doi:10.1371/journal.pntd.0014067)
Supplement: Supplementary file 1 [file pntd.0014067.s010.docx]

Dear Editor,

On behalf of all co-authors, I would like to express our gratitude for the opportunity to revise and resubmit our manuscript titled, "New insights on the Feeding Site and Salivation by *Lutzomyia longipalpis* (Diptera: Psychodidae) During Blood Ingestion on Host Skin". We appreciate the time and effort that you and the reviewers have devoted to evaluating our work. We are confident that suggestions have enhanced both the quality and clarity of the document.

We have thoroughly addressed all comments and suggestions provided by the reviewers and have made the necessary revisions to the manuscript. A comprehensive, point-by-point response to each reviewer's comment has been prepared below. In this response, we outline the modifications made to the manuscript and their locations (*in red*) according to the file “Manuscript”. All revisions within the manuscript have been distinctly highlighted to aid in the review process and submitted as "Revised manuscript with track changes". We hope that the updated manuscript aligns with the journal standards, and we eagerly await your decision.

Thank you for your time and consideration.

Sincerely,

Kelsilandia Aguiar Martins

**Journal Requirements:**

If the reviewer comments include a recommendation to cite specific previously published works, please review and evaluate these publications to determine whether they are relevant and should be cited. There is no requirement to cite these works unless the editor has indicated otherwise.

1) We ask that a manuscript source file is provided at Revision. Please upload your manuscript file as a .doc, .docx, .rtf or .tex. If you are providing a .tex file, please upload it under the item type u2018LaTeX Source Fileu2019 and leave your .pdf version as the item type u2018Manuscriptu2019.
2) Your manuscript's sections are not in the correct order. Please amend to the following order: Abstract, Introduction, Results, Discussion, and Methods
3) We have noticed that you have uploaded Supporting Information files, but you have not included a list of legends. Please add a full list of legends for your Supporting Information files after the references list.
4) We are unable to open the following Supporting Information files: S1 Video.avi, S4 Video.avi, and S5 Video.avi. Please kindly revise as necessary and re-upload.

1-4) We have revised the manuscript in accordance with the editor's recommendations, and the changes have been incorporated regarding the suggestions.

5) We note that your Data Availability Statement is currently as follows: "All data are accessible to readers". Please confirm at this time whether or not your submission contains all raw data required to replicate the results of your study. Authors must share the “minimal data set” for their submission. PLOS defines the minimal data set to consist of the data required to replicate all study findings reported in the article, as well as related metadata and methods (https://journals.plos.org/plosone/s/data-availability#loc-minimal-data-set-definition). For example, authors should submit the following data:

The values behind the means, standard deviations and other measures reported;

The values used to build graphs;

The points extracted from images for analysis..

Authors do not need to submit their entire data set if only a portion of the data was used in the reported study. If your submission does not contain these data, please either upload them as Supporting Information files or deposit them to a stable, public repository and provide us with the relevant URLs, DOIs, or accession numbers. For a list of recommended repositories, please see https://journals.plos.org/plosone/s/recommended-repositories.

If there are ethical or legal restrictions on sharing a de-identified data set, please explain them in detail (e.g., data contain potentially sensitive information, data are owned by a third-party organization, etc.) and who has imposed them (e.g., an ethics committee). Please also provide contact information for a data access committee, ethics committee, or other institutional body to which data requests may be sent. If data are owned by a third party, please indicate how others may request data access.

5)We have included a supplementary file (S1 Dataset) covering the missing data cited in the text

6) Please amend your detailed Financial Disclosure statement. This is published with the article. It must therefore be completed in full sentences and contain the exact wording you wish to be published.

1) State the initials, alongside each funding source, of each author to receive each grant. For example: "This work was supported by the National Institutes of Health (####### to AM; ###### to CJ) and the National Science Foundation (###### to AM)."

"The funders had no role in study design, data collection and analysis, decision to publish, or preparation of the manuscript."

State what role the funders took in the study. If the funders had no role in your study, please state: "The funders had no role in study design, data collection and analysis, decision to publish, or preparation of the manuscript." 3) If any authors received a salary from any of your funders, please state which authors and which funders..

If you did not receive any funding for this study, please simply state: u201cThe authors received no specific funding for this work.u201d

7) Your current Financial Disclosure states, "Yes ↳ Please add funding details. Financial support: Coordenação de

Aperfeiçoamento de Pessoal de Nível Superior (CAPES), Fundação de Amparo à Pesquisa do Estado de Minas

Gerais (FAPEMIG), Conselho Nacional de Desenvolvimento Científico e Tecnológico (CNPq) and Instituto Nacional de Ciência e Tecnologia em Entomologia Molecular (INCT EM). ↳ Please select the country of your main research funder

(please select carefully as in some cases this is used in fee calculation). BRAZIL - BR".

However, your funding information on the submission form indicates missing the fund from Instituto Nacional de Ciência e Tecnologia em Entomologia Molecular (INCT EM)..

Please indicate by return email the full and correct funding information for your study and confirm the order in which funding contributions should appear. Please be sure to indicate whether the funders played any role in the study design, data collection and analysis, decision to publish, or preparation of the manuscript.

6-7) Amendments were performed in our Financial Disclosure. It was previously covering all sources of finances involved (including scholarships), We left the main grant designed for this project, including its reference number.

8) Some material included in your submission may be copyrighted. According to PLOS's copyright policy, authors who use figures or other material (e.g., graphics, clipart, maps) from another author or copyright holder must demonstrate or obtain permission to publish this material under the Creative Commons Attribution 4.0 International (CC BY 4.0) License used by PLOS journals. Please closely review the details of PLOS's copyright requirements here: [PLOS](https://journals.plos.org/plosntds/s/licenses-and-copyright)

[Licenses and Copyright.](https://journals.plos.org/plosntds/s/licenses-and-copyright) If you need to request permissions from a copyright holder, you may use the [PLOS Content Copyright Permission Form](https://storage.googleapis.com/plos-published-prod/c4ff/Content%20Copyright%20Permission%20Form.pdf?X-Goog-Algorithm=GOOG4-RSA-SHA256&X-Goog-Credential=wombat-sa%40plos-prod.iam.gserviceaccount.com%2F20220817%2Fauto%2Fstorage%2Fgoog4_request&X-Goog-Date=20220817T210608Z&X-Goog-Expires=86400&X-Goog-SignedHeaders=host&X-Goog-Signature=cd9dc868773378f7033b9f8cfb91430167d6275df90bce01342a454f36512530b64f24736fbf68c4fd96da74d663b6ee95b3420bded8fb0e833af2390b896b8706643b476d7f532408921b60576d65b60617070900fd2bcd28eec4cd32fefcf007d3655df17fcc91023329a28479ce0d1c5758174eedcc9e3c1788c5f7dd8523f3ddd871e02c998a20f7dc78ec00f313d4b5a78ba2037a6c7ef0a05dc29817da9368065460c8d8446659f35f0458fefabd3101eb7f926e9cb7a0b53e7bbe47aaed3e0ba52f9af9c2b8f6d7d3a8871b205fc4cbe97384ed37c7604fd7eecfedccf49cba12820b2e3b649541cefde3103cd7d72731b3e8b0248439b4cba691ca8a) Please respond directly to this email and provide any known details concerning your material's license terms and permissions required for reuse, even if you have not yet obtained copyright permissions or are unsure of your material's copyright compatibility. Once you have responded and addressed all other outstanding technical requirements, you may resubmit your manuscript within Editorial Manager. Potential Copyright Issues:

i) Please confirm (a) that you are the photographer of 2, or (b) provide written permission from the photographer to publish the photo(s) under our CC BY 4.0 license.
ii) Please confirm whether you drew the images / clip-art within the figure panels by hand. If you did not draw the images, please provide (a) a link to the source of the images or icons and their license / terms of use; or (b) written permission from the copyright holder to publish the images or icons under our CC BY 4.0 license. Alternatively, you may replace the images with open source alternatives. See these open source resources you may use to replace images / clip-art: - https://commons.wikimedia.org - <https://openclipart.org/>

8)The Figure 1 from the manuscript (currently figure 7) has illustrations done using the Biorender software. The license was attached during submission and all photos included in this manuscript were performed by the main author. All photos or videos in the current manuscript were performed by Kelsilandia Aguiar Martins.

**Reviewers' Comments:**

**Methods**

Reviewer #3: The objective of this study was to characterize the mechanical actions of Lutzomyia longipalpis mouthparts and the patterns of salivary secretion during blood feeding on host skin. The study is primarily descriptive, using intravital microscopy to evaluate feeding behavior and salivation dynamics during the probing and feeding phases. While the descriptive nature of the work is valuable and novel, the methodology requires further clarification.

Regarding the methodology, I have two major comments:

First, the manuscript does not clearly state the total number of mice or sand flies used in each experiment. It is also unclear whether animals were used multiple times with different sand flies, or what the total number of recorded feeding events and observations was. To strengthen the results, please provide the sample size "n" for both animals and individual feeding observations. In addition, please clarify whether a single fly was used per ear or if multiple flies were tested.

Thank you for reminding us of such important details. We have included a supplementary file (S1 Dataset) and included the information on results and method (lines 125-130) and (545-547)

Second, the authors measure the pH of the salivary glands of Lutzomyia longipalpis, however, the relevance of this result is not clearly discussed in the manuscript. Further clarification of the experimental design in the Methods section is needed. Specifically, it is unclear whether the salivary glands were obtained from sand flies treated with Acridine Orange or from untreated controls. The authors should also clarify whether pH differences were evaluated between treated and untreated groups.

-We agree with the reviewer and included the following setence on methods (lines 513-514)

-To avoid any potential misunderstanding, we have reordered the Methods section by moving the subsection entitled "Measurement of Salivary Gland pH" so that it precedes "Saliva Labeling."

**Results**

Reviewer #2: The authors need to double-check the figure labels with the figure legends as they did not always match.

Thank you, we have reviewed carefully the citation of the figures.

Reviewer #3: Overall, the first part of the Results is well supported by images and videos. However, important methodological details and clarity in data presentation are lacking. A better balance between description and visual documentation would improve the section.

- The statement “during which small blood vessels became temporarily invisible under microscopic observation” is not supported by any referenced figure or video. It is unclear how this observation was determined, and no visual evidence is provided. The authors should include a representative image or video or revise the statement for clarity.

-We revised the passage below and included an additional video (Video S3) in the manuscript to better illustrate the reestablishment of blood flow toward the insect's mouthparts.

- Similarly, the description of the gradual reestablishment of blood flow in one or more vessels (Figures 2G and 2H) is not clearly illustrated.

- Please see the previous answer. The same video can be applied to both questions.

- Line 232: Should this refer to Figures 2F and 2H instead?

- Yes, the observation is correct and amended in the text (Line 162)

- In Figure 3, vasodilation should be explicitly indicated in the image. In addition, the “prominence of blood” described in Figure 3D is ambiguous, please clarify whether this represents hemorrhage or vasodilation.

- Arrows have been added to the images in Figure 3B and D (now figure 2) to clearly show “typical vasoconstriction” (B) and “pronounced vasodilation” (D).

- Lines 267-270 and 270-272: The authors describe experiments regarding salivary gland labeling after sucrose feeding (n=10) and survival rates (n=30). However, no supporting data (table or figure) are presented. These data should be included, at least as Supplementary Material.

We agree and a supplementary file was included with the information (S1 Datafile)

- In the section “Salivation by Lutzomyia longipalpis in the Host Skin,” the authors describe key observations, including: (i) visualization of salivary release immediately after biting and throughout feeding, (ii) saliva exiting near the tip of the mouthparts, and (iii) a rapid transition of saliva to a gel-like consistency upon exposure to air. However, the first two paragraphs are not supported by referenced figures or videos. Later in the manuscript, similar descriptions are repeated, this time with figure references. The authors should consolidate these descriptions and ensure that all key observations are directly supported by visual evidence at their first mention.

- We agree with the reviewer and have incorporated references and videos in the paragraph to better illustrate the salivation pattern of *Lu. longipalpis* on the host skin (229-235)

- In this section, the use of “above” and “below” (Lines 307–308) is unclear. To improve clarity, I suggest subdividing Figure 5A into labeled panels, such as: (i) original 8-bit images, (ii) feeding site and processed output obtained via frame subtraction during the initial feeding phase, and (iii–iv) corresponding views during subsequent feeding phases.
- Line 315: Should this refer to 10 sequential time points? As currently described, the upper panels correspond to original 8-bit images, while the lower panels show processed images generated by frame subtraction using the Image Calculator tool.

-We agree with the reviewer and have revised the legend for Figure 5 (now Figure 4) to improve its clarity.

- The observation of “gel-like saliva,” along with Supplementary Video S6, is discussed only in the Discussion section but should be presented in the Results. In addition, the authors should report the number of observed events and indicate whether this phenomenon was assessed in both treated and untreated groups with Acridine Orange.

The gel-like saliva was an important observation made when the insect's mouthparts penetrated the skin. We decided to keep this observation primarily in the Discussion section, as it is interesting to reflect on the biological properties of the saliva; however, this scenario is unlikely to accurately represent typical sand fly feeding behavior. In natural environments, penetration of the mouthparts through the skin would likely occur only in areas where the skin is especially thin. Therefore, this observation is more indicative of the experimental conditions than a true representation of the feeding process.

- Lines 481–484; 508–510; 515–520: These paragraphs include strong statements that are not supported by references. Appropriate citations should be added.

References were included to support the statements (Line 438-443, 464-471, 472-477)

**Conclusions**

Reviewer #3: Regarding the final statement of the Conclusion (Lines 535-537) the authors claim that their findings provide new insights into how feeding behavior enhances parasite transmission and highlight salivary factors as key targets for transmission-blocking strategies. However, the current Discussion does not sufficiently bridge the gap between the descriptive results and the actual mechanisms of parasite transmission. Moreover, the manuscript lacks functional evidence or targeted experiments to support these broad claims.

We took great care in removing this sentence to ensure that we do not overextend the significance of our results.

Reviewer #2: The authors need to double-check the figure labels with the figure legends as they did not always match.

We agree with the reviewer and have revised the figure`s citations

- Lines 41 and 120: Please correct the typographical errors identified in these lines.

- The typographical errors identified in these lines (41 and 120) were corrected:

L.41: “activation of small dermal ~~"feeder vessels."~~ **"feeder vessels".**

L.120:. “Although the mechanical aspects of blood acquisition and the role of saliva in ~~Leishmania~~ ***Leishmania***”

We have addressed all these corrections (L39 and 119)

- Figure 2 labeling: There is an inconsistency in the acronyms used for “mouthparts.” The figure uses “Mp,” whereas the legend refers to “Mo.” Please standardize this terminology throughout the manuscript and figures.

The abbreviation for mouthparts has been standardized as Mp. All figure labels and corresponding legends have been revised throughout the manuscript to ensure consistency.

Line 156: Please provide the full definition of the acronym “IS” at its first occurrence.

- IS is the abbreviation for insect saline. This abbreviation first appears on line 518).

- Line 206: It is recommended to cite Video S1 alongside the figures mentioned in this section, as the video offers a more representative and dynamic view of the phenomenon than the figure.

-We agree with the reviewer and have cited the new Video S2 together with Figs. 2A and 2B to illustrate the characteristic “scissor-like” movements of the mouthparts. (Line 143-144)

- Figure 3 (line 259): In the image, the authors do not show any representation of vasoconstriction. Although this may be clear, they should either indicate vasoconstriction in the figure or remove this from the legend.

- Arrows have been added to the images in (now Figure 2) to clearly show “typical vasoconstriction” (B) and “pronounced vasodilation” (D).

- Figure 6B: Please include clear annotations or markers in the image corresponding to the descriptions provided in the legend (Lines 371–373). This will help readers identify the specific structures mentioned.

The images in (now Figure 5B) have been edited, and the arrows mentioned in the legend have been included.

- Video S3 legend: The legend currently refers to “red and white arrows”; however, these markers are not present in the video. Please revise the legend for accuracy or add the missing markers to the video.

-The reviewer is correct. We inadvertently uploaded an earlier version of S3 Video prior to the final editing, which did not include all of the annotated markers described in the video legend. We have now replaced it with the correct version, which contains all of the markers referenced in the caption (Please note its now referred as S4 Video)

**Summary and General Comments**

Reviewer #1: The manuscript by Martins et al. presents interesting and novel insights into the blood-feeding behavior of Lutzomyia longipalpis on its host. The data are well presented and appropriately discussed. Most figures are clear and effectively illustrate the results.The only exception is Figure 2, which requires revision. Vasodilation of the “feeding vessel” is clearly depicted in Figure 2E and 2F, whereas the remainder of the figure is either of relatively low quality (C and D) or difficult to interpret. I suggest either moving the entire Figure 2 to the Supplementary Materials or retaining only the two images that clearly depict vasodilation of the “feeding vessel.”

We have included a video from the same figure to help with the interpretation

In Figure 3, there is a minor error: although vascular alteration is clearly demonstrated, vasoconstriction (Vc) is not labeled in Figure 3B.

- Arrows have been added to the images in (now Figure 2B and D) to clearly show “typical vasoconstriction” (B) and “pronounced vasodilation” (D).

Finally, I would like to comment on the concept of a “one gland with two acini” (see lines 152–154). Two salivary glands represent the standard anatomical arrangement in most Diptera, including mosquitoes, black flies, and biting midges. Similarly, sand flies also possess two salivary glands. The fact that these glands drain into a common salivary duct does not justify referring to them as a single gland. Mosquitoes likewise have a pair of salivary glands (left and right), each with its own lateral salivary duct. These ducts join to form a single common salivary duct, which then opens into the hypopharynx. In summary, the correct anatomical arrangement in sand flies consists of paired glands, paired ducts, and a common salivary duct. I kindly ask the authors to adopt this terminology and revise the manuscript accordingly.

- The reviewer is correct. The sand fly salivary glands (SGs) are paired organs. Each gland typically consists of a single rounded lobe. Accordingly, we have revised the sentence below and replaced the terms "acinus" and "acini" with "lobe" and "lobes", respectively, throughout the manuscript.

Reviewer #2:

Points to address:

Line 56: Phlebotomine sand flies are the only proven vector of Leishmania infantum - use of the word 'main' suggests that there are other vectors.

-The word “main” was replaced by "principal" in the sentence (Line 55) below:

Line 63. Explain/qualify what vessel 'recruitment' is in the context of your results.

The central idea of "feeder vessels" is that the sand fly appears to create a partially ruptured but still functioning microvessel. Rather than completely destroying the vessel, mouthparts break the wall vessel while leaving it connected to the circulation. The vessel continues to carry blood, can still undergo vasoconstriction under the suction of the insect, and provides a continuous, localized blood source. This interpretation also explains how saliva can enter the vessel lumen despite mechanical disruption of the vessel wall. We propose that these "feeder vessels" are injured but patent microvessels, representing a specialized vascular state that facilitates efficient blood feeding.

To clarify the nature of these "feeder vessels”, we include an extra paragraph in the discussion (362-380):

Line 86/87: Add a diagram here of the female sand fly head with labels of the different mouthparts to allow the reader to interpret the paper more easily.

We included an illustration in figure 7 to help to visualize the mouthparts position in relation to the "feeding vessel".

Line 119: Add Peters et al. 2008 (Nathan C. Peters et al. ,In Vivo Imaging Reveals an Essential Role for Neutrophils in Leishmaniasis Transmitted by Sand Flies.Science321,970-974(2008).DOI:10.1126/science.1159194) and

Giraud et al.2018 (Giraud E, Lestinova T, Derrick T, Martin O, Dillon RJ, Volf P, Műller I, Bates PA, Rogers ME. Leishmania proteophosphoglycans regurgitated from infected sand flies accelerate dermal wound repair and exacerbate leishmaniasis via insulin-like growth factor 1-dependent signalling. PLoS Pathog. 2018 Jan 19;14(1):e1006794. doi: 10.1371/journal.ppat.1006794. PMID: 29352310; PMCID: PMC5792026). - these paper identifiy the interaction of uninfected and infected sand fly bites with the wound healing processes of the host, which are exploited by Leishmania.

The references above were included as sugested.

Line 177: Figure 1 - add dimensions to the customised feeding chamber.

The dimensions of the feeding chamber have been included on methods (line 537)

Line 264: add "crop" in brackets after diverticulum to make it clearer.

It was included as suggested (line 202)

-Line 264: “…resulted in strong and efficient fluorescence labeling of both the diverticulum **(crop)** and the contentes…”

Line 272: add this survival data to the supplementary information.

The data was included as S1 Dataset file.

Line 279: Figure 4 - the acinus in panel F has released its saliva - please replace with a picture of one that remains intact, in which the intraluminal saliva can be observed (stained with bromocresol purple) - for comparison with panel E, stained with bromothymol blue.

We respectfully believe that repeating the experiment is not necessary, but we need probably to clarify better the experiment done. The pH determination did not rely on visualization or staining of an intact salivary gland lumen. It was perhaps not clear enough during our writing, and we tried to improve in the new version.

As better described now in the Methods, individual lobes were transferred to a saturated solution of the pH indicator, after which they were deliberately ruptured. The pH was determined from the color change that occurred when the released luminal contents mixed with the external indicator solution. Therefore, mechanical rupture of the acinus constitutes an essential step of the experimental procedure rather than an experimental artefact. The images shown in Figure 4 (now Figure 3) are representative illustrations of the color transition observed after release of the gland contents and are not themselves the basis for the pH determination. Likewise, the purpose of panels C and D is simply to illustrate the distinct color responses obtained with the two pH indicators following release of the luminal contents. Whether the acinus appears partially depleted or remains morphologically intact in the image does not influence interpretation of the pH measurement, because the assay depends on mixing the luminal fluid with the external indicator solution after mechanical rupture, rather than on visualization of saliva retained within the gland lumen. Both panels depict the same experimental endpoint, namely the color of the indicator immediately after mixing with the released luminal contents. Consequently, the degree of morphological preservation of the acinus is not relevant to interpretation of the assay.

Lines 425-428: Can you give some idea of how long the sandfly was engaged in these different phases of blood feeding?

We have analysed some recordings on bright field where females could be tracked since the landing on skin, until their leave after their engorgement. The data from these observations can be found in S1 Dataset.

Line 428: Can you explain what you mean by recruitment of vessels and if this has been observed for other vectors or in other contexts as I am unclear how this (physiologically) happens.

-Although the feeding mechanism (telmophagy) is described in both *Ornithodoros rostratus* and *Lu. longipalpis* relies on blood acquisition through disruption of the dermal microvasculature and the establishment of an extravascular hemorrhagic microenvironment; the mechanisms underlying vessel disruption differ substantially. In ticks, vascular damage is primarily mediated by the cytotoxic activity of saliva. In contrast, in phlebotomine sand flies, the mouthparts play the initial mechanical role in vessel rupture, aided by the bioactivity of this saliva to maintain the vessel without collapsing it. To the best of our knowledge, however, no previous study has described a mechanism comparable to the "feeder vessels" observed at the feeding site of hematophagous arthropods

Accordingly, we have revised the explanation in the Discussion section to improve its clarity.

Line 432: Did you observe any sand flies to directly cannulate a vessel for feeding. If so, what proportion of flies do this and for how long?

-We devoted considerable effort to determining whether sand flies feed directly from blood vessels (especially after watching the saliva inside the blood vessels). During our initial experiments, we repeatedly changed objective lenses and continuously adjusted the focal plane in an attempt to visualize this process, but without success. Only after these preliminary observations did we adopt a more stable imaging approach, avoiding frequent changes in magnification and focus, which ultimately allowed us to characterize the feeding behavior of the insects.

Our observations suggest that, although the insect mechanically ruptures a blood vessel and induces local hemorrhage, it does not suck the blood directly inside the newly ruptured vessel. Instead, blood appears to flow through the surrounding tissue before reaching the insect's mouthparts. **Conversely,** we observed several situations during probing, where there was a“pool of blood” formed after a rupture of vessels, but the insect still moved to other areas where “feeder vessels” were selected. We added a new figure and illustration to the material and methods section that could help to visualise it better. All the insects recorded presented the same behaviour, which gives us confidence about the description of their feeding process.

Reviewer #3: Overall, while the study provides interesting and novel observations on the feeding behavior of Lutzomyia longipalpis using intravital microscopy. Several important aspects require clarification to strengthen the manuscript, in particular, key methodological details are missing, including sample sizes, experimental design, and the number of observed events, which limits reproducibility and interpretation of the results. Also, while the findings are insightful, certain limitations should be addressed in the discussion, such as the study was conducted in the absence of Leishmania parasites, as the presence of the parasite and its associated PSG is known to significantly modify sand fly feeding mechanics.

Hopefully, we addressed all the questions and requirements from reviewers to make our manuscript clearer and more robust. We also performed experiments with *Leishmania*, however; we decided to dedicate this paper exclusively to describing the feeding process and salivation pattern. Indeed, the presence of the parasite affected insect behavior, as suggested.
