# Supplementary material for: New insights on the Feeding Site and Salivation by Lutzomyia longipalpis (Diptera: Psychodidae) During Blood Ingestion on Host Skin
Source: PLoS Negl Trop Dis. 2026 Aug 24;20(8):e0014067. doi: 10.1371/journal.pntd.0014067 (PMC13568499; doi:10.1371/journal.pntd.0014067)
Supplement: Peer review history

# Peer review: Decision Letter 0

**DOI:** 10.1371/journal.pntd.0014067.r001

**Authors:** Eric Calvo, Audrey Lenhart

28 Apr 2026

New insights on the Feeding Site and Salivation by Lutzomyia longipalpis (Diptera: Psychodidae) During Blood Ingestion on Host Skin

PLOS Neglected Tropical Diseases

Dear Dr. Aguiar Martins,

Thank you for submitting your manuscript to PLOS Neglected Tropical Diseases. After careful consideration, we feel that it has merit but does not fully meet PLOS Neglected Tropical Diseases's publication criteria as it currently stands. Therefore, we invite you to submit a revised version of the manuscript that addresses the points raised during the review process.

Please submit your revised manuscript within by Jun 27 2026 11:59PM. If you will need more time than this to complete your revisions, please reply to this message or contact the journal office at plosntds@plos.org. When you're ready to submit your revision, log on to https://www.editorialmanager.com/pntd/ and select the 'Submissions Needing Revision' folder to locate your manuscript file.

Please include the following items when submitting your revised manuscript:

* A letter that responds to each point raised by the editor and reviewer(s). You should upload this letter as a separate file labeled 'Response to Reviewers'. This file does not need to include responses to any formatting updates and technical items listed in the 'Journal Requirements' section below.

* A marked-up copy of your manuscript that highlights changes made to the original version. You should upload this as a separate file labeled 'Revised Manuscript with Track Changes'.

* An unmarked version of your revised paper without tracked changes. You should upload this as a separate file labeled 'Manuscript'.

If you would like to make changes to your financial disclosure, competing interests statement, or data availability statement, please make these updates within the submission form at the time of resubmission. Guidelines for resubmitting your figure files are available below the reviewer comments at the end of this letter.

We look forward to receiving your revised manuscript.

Kind regards,

Eric Calvo

Academic Editor

PLOS Neglected Tropical Diseases

Audrey Lenhart

Section Editor

PLOS Neglected Tropical Diseases

Shaden Kamhawi

co-Editor-in-Chief

PLOS Neglected Tropical Diseases

orcid.org/0000-0003-4304-636XX

Paul Brindley

co-Editor-in-Chief

PLOS Neglected Tropical Diseases

orcid.org/0000-0003-1765-0002

**Journal Requirements:**

If the reviewer comments include a recommendation to cite specific previously published works, please review and evaluate these publications to determine whether they are relevant and should be cited. There is no requirement to cite these works unless the editor has indicated otherwise.

1) We ask that a manuscript source file is provided at Revision. Please upload your manuscript file as a .doc, .docx, .rtf or .tex. If you are providing a .tex file, please upload it under the item type u2018LaTeX Source Fileu2019 and leave your .pdf version as the item type u2018Manuscriptu2019.

2) Your manuscript's sections are not in the correct order.  Please amend to the following order: Abstract, Introduction, Results, Discussion, and Methods

3) We have noticed that you have uploaded Supporting Information files, but you have not included a list of legends. Please add a full list of legends for your Supporting Information files after the references list.

4) We are unable to open the following Supporting Information files: S1 Video.avi, S4 Video.avi, and S5 Video.avi. Please kindly revise as necessary and re-upload.

5) We note that your Data Availability Statement is currently as follows: "All data are accessible to readers". Please confirm at this time whether or not your submission contains all raw data required to replicate the results of your study. Authors must share the “minimal data set” for their submission. PLOS defines the minimal data set to consist of the data required to replicate all study findings reported in the article, as well as related metadata and methods (https://journals.plos.org/plosone/s/data-availability#loc-minimal-data-set-definition).

For example, authors should submit the following data:

1) The values behind the means, standard deviations and other measures reported;

2) The values used to build graphs;

3) The points extracted from images for analysis..

Authors do not need to submit their entire data set if only a portion of the data was used in the reported study.

If your submission does not contain these data, please either upload them as Supporting Information files or deposit them to a stable, public repository and provide us with the relevant URLs, DOIs, or accession numbers. For a list of recommended repositories, please see https://journals.plos.org/plosone/s/recommended-repositories.

If there are ethical or legal restrictions on sharing a de-identified data set, please explain them in detail (e.g., data contain potentially sensitive information, data are owned by a third-party organization, etc.) and who has imposed them (e.g., an ethics committee). Please also provide contact information for a data access committee, ethics committee, or other institutional body to which data requests may be sent. If data are owned by a third party, please indicate how others may request data access.

6) Please amend your detailed Financial Disclosure statement. This is published with the article. It must therefore be completed in full sentences and contain the exact wording you wish to be published.

1) State the initials, alongside each funding source, of each author to receive each grant. For example: "This work was supported by the National Institutes of Health (####### to AM; ###### to CJ) and the National Science Foundation (###### to AM)."

2) State what role the funders took in the study. If the funders had no role in your study, please state: "The funders had no role in study design, data collection and analysis, decision to publish, or preparation of the manuscript."

3) If any authors received a salary from any of your funders, please state which authors and which funders..

If you did not receive any funding for this study, please simply state: u201cThe authors received no specific funding for this work.u201d

7) Your current Financial Disclosure states, "Yes ↳ Please add funding details. Financial support: Coordenação de Aperfeiçoamento de Pessoal de Nível Superior (CAPES), Fundação de Amparo à Pesquisa do Estado de Minas Gerais (FAPEMIG), Conselho Nacional de Desenvolvimento Científico e Tecnológico (CNPq) and Instituto Nacional de Ciência e Tecnologia em Entomologia Molecular (INCT EM). ↳ Please select the country of your main research funder (please select carefully as in some cases this is used in fee calculation). BRAZIL - BR".

However, your funding information on the submission form indicates missing the fund from Instituto Nacional de Ciência e Tecnologia em Entomologia Molecular (INCT EM)..

Please indicate by return email the full and correct funding information for your study and confirm the order in which funding contributions should appear. Please be sure to indicate whether the funders played any role in the study design, data collection and analysis, decision to publish, or preparation of the manuscript.

8) Some material included in your submission may be copyrighted. According to PLOS's copyright policy, authors who use figures or other material (e.g., graphics, clipart, maps) from another author or copyright holder must demonstrate or obtain permission to publish this material under the Creative Commons Attribution 4.0 International (CC BY 4.0) License used by PLOS journals. Please closely review the details of PLOS's copyright requirements here: PLOS Licenses and Copyright. If you need to request permissions from a copyright holder, you may use the PLOS Content Copyright Permission Form Please respond directly to this email and provide any known details concerning your material's license terms and permissions required for reuse, even if you have not yet obtained copyright permissions or are unsure of your material's copyright compatibility. Once you have responded and addressed all other outstanding technical requirements, you may resubmit your manuscript within Editorial Manager. Potential Copyright Issues:

i) Please confirm (a) that you are the photographer of 2, or (b) provide written permission from the photographer to publish the photo(s) under our CC BY 4.0 license.

ii) 1.: Please confirm whether you drew the images / clip-art within the figure panels by hand. If you did not draw the images, please provide (a) a link to the source of the images or icons and their license / terms of use; or (b) written permission from the copyright holder to publish the images or icons under our CC BY 4.0 license. Alternatively, you may replace the images with open source alternatives. See these open source resources you may use to replace images / clip-art: - https://commons.wikimedia.org - https://openclipart.org/

**Reviewers' Comments:**

Reviewer's Responses to Questions

**Key Review Criteria Required for Acceptance?**

As you describe the new analyses required for acceptance, please consider the following:

**Methods**

-Are the objectives of the study clearly articulated with a clear testable hypothesis stated?

-Is the study design appropriate to address the stated objectives?

-Is the population clearly described and appropriate for the hypothesis being tested?

-Is the sample size sufficient to ensure adequate power to address the hypothesis being tested?

-Were correct statistical analysis used to support conclusions?

-Are there concerns about ethical or regulatory requirements being met?

Reviewer #1: Study design is appropriate to address the stated objectives. Sample size is sufficient to address the hypothesis.

Reviewer #2: The methods are innovative and appropriate. The study design was appropriate with enough samples to evidence their observations.

Reviewer #3: The objective of this study was to characterize the mechanical actions of Lutzomyia longipalpis mouthparts and the patterns of salivary secretion during blood feeding on host skin. The study is primarily descriptive, using intravital microscopy to evaluate feeding behavior and salivation dynamics during the probing and feeding phases. While the descriptive nature of the work is valuable and novel, the methodology requires further clarification.

Regarding the methodology, I have two major comments:

First, the manuscript does not clearly state the total number of mice or sand flies used in each experiment. It is also unclear whether animals were used multiple times with different sand flies, or what the total number of recorded feeding events and observations was. To strengthen the results, please provide the sample size "n" for both animals and individual feeding observations. In addition, please clarify whether a single fly was used per ear or if multiple flies were tested.

Second, the authors measure the pH of the salivary glands of Lutzomyia longipalpis, however, the relevance of this result is not clearly discussed in the manuscript. Further clarification of the experimental design in the Methods section is needed. Specifically, it is unclear whether the salivary glands were obtained from sand flies treated with Acridine Orange or from untreated controls. The authors should also clarify whether pH differences were evaluated between treated and untreated groups.

**********

**Results**

-Does the analysis presented match the analysis plan?

-Are the results clearly and completely presented?

-Are the figures (Tables, Images) of sufficient quality for clarity?

Reviewer #1: The results are clearly and completely presented. For quality and clarity of figures see my comments to the authors.

Reviewer #2: The authors need to double-check the figure labels with the figure legends as they did not always match.

Reviewer #3: Overall, the first part of the Results is well supported by images and videos. However, important methodological details and clarity in data presentation are lacking. A better balance between description and visual documentation would improve the section.

- The statement “during which small blood vessels became temporarily invisible under microscopic observation” is not supported by any referenced figure or video. It is unclear how this observation was determined, and no visual evidence is provided. The authors should include a representative image or video or revise the statement for clarity.

- Similarly, the description of the gradual reestablishment of blood flow in one or more vessels (Figures 2G and 2H) is not clearly illustrated.

- Line 232: Should this refer to Figures 2F and 2H instead?

- In Figure 3, vasodilation should be explicitly indicated in the image. In addition, the “prominence of blood” described in Figure 3D is ambiguous, please clarify whether this represents hemorrhage or vasodilation.

- Lines 267-270 and 270-272: The authors describe experiments regarding salivary gland labeling after sucrose feeding (n=10) and survival rates (n=30). However, no supporting data (table or figure) are presented. These data should be included, at least as Supplementary Material.

- In the section “Salivation by Lutzomyia longipalpis in the Host Skin,” the authors describe key observations, including: (i) visualization of salivary release immediately after biting and throughout feeding, (ii) saliva exiting near the tip of the mouthparts, and (iii) a rapid transition of saliva to a gel-like consistency upon exposure to air. However, the first two paragraphs are not supported by referenced figures or videos. Later in the manuscript, similar descriptions are repeated, this time with figure references. The authors should consolidate these descriptions and ensure that all key observations are directly supported by visual evidence at their first mention.

- In this section, the use of “above” and “below” (Lines 307–308) is unclear. To improve clarity, I suggest subdividing Figure 5A into labeled panels, such as: (i) original 8-bit images, (ii) feeding site and processed output obtained via frame subtraction during the initial feeding phase, and (iii–iv) corresponding views during subsequent feeding phases.

- Line 315: Should this refer to 10 sequential time points? As currently described, the upper panels correspond to original 8-bit images, while the lower panels show processed images generated by frame subtraction using the Image Calculator tool.

- The observation of “gel-like saliva,” along with Supplementary Video S6, is discussed only in the Discussion section but should be presented in the Results. In addition, the authors should report the number of observed events and indicate whether this phenomenon was assessed in both treated and untreated groups with Acridine Orange.

- Lines 481–484; 508–510; 515–520: These paragraphs include strong statements that are not supported by references. Appropriate citations should be added.

**********

**Conclusions**

-Are the conclusions supported by the data presented?

-Are the limitations of analysis clearly described?

-Do the authors discuss how these data can be helpful to advance our understanding of the topic under study?

-Is public health relevance addressed?

Reviewer #1: Conclusions are supported by the data and authors discuss how these data can be helpful to advance our understanding of the topic.

Reviewer #2: Yes.

Reviewer #3: Regarding the final statement of the Conclusion (Lines 535-537) the authors claim that their findings provide new insights into how feeding behavior enhances parasite transmission and highlight salivary factors as key targets for transmission-blocking strategies. However, the current Discussion does not sufficiently bridge the gap between the descriptive results and the actual mechanisms of parasite transmission. Moreover, the manuscript lacks functional evidence or targeted experiments to support these broad claims.

**********

**Editorial and Data Presentation Modifications?**

Use this section for editorial suggestions as well as relatively minor modifications of existing data that would enhance clarity. If the only modifications needed are minor and/or editorial, you may wish to recommend “Minor Revision” or “Accept”.

Reviewer #1: minor revision

Reviewer #2: The authors need to double-check the figure labels with the figure legends as they did not always match.

Reviewer #3: - Lines 41 and 120: Please correct the typographical errors identified in these lines.

- Figure 2 labeling: There is an inconsistency in the acronyms used for “mouthparts.” The figure uses “Mp,” whereas the legend refers to “Mo.” Please standardize this terminology throughout the manuscript and figures.

- Line 156: Please provide the full definition of the acronym “IS” at its first occurrence.

- Line 206: It is recommended to cite Video S1 alongside the figures mentioned in this section, as the video offers a more representative and dynamic view of the phenomenon than the figure.

- Figure 3 (line 259): In the image, the authors do not show any representation of vasoconstriction. Although this may be clear, they should either indicate vasoconstriction in the figure or remove this from the legend.

- Figure 6B: Please include clear annotations or markers in the image corresponding to the descriptions provided in the legend (Lines 371–373). This will help readers identify the specific structures mentioned.

- Video S3 legend: The legend currently refers to “red and white arrows”; however, these markers are not present in the video. Please revise the legend for accuracy or add the missing markers to the video.

**********

**Summary and General Comments**

Use this section to provide overall comments, discuss strengths/weaknesses of the study, novelty, significance, general execution and scholarship. You may also include additional comments for the author, including concerns about dual publication, research ethics, or publication ethics. If requesting major revision, please articulate the new experiments that are needed.

Reviewer #1: The manuscript by Martins et al. presents interesting and novel insights into the blood-feeding behavior of Lutzomyia longipalpis on its host. The data are well presented and appropriately discussed. Most figures are clear and effectively illustrate the results.

The only exception is Figure 2, which requires revision. Vasodilation of the “feeding vessel” is clearly depicted in Figure 2E and 2F, whereas the remainder of the figure is either of relatively low quality (C and D) or difficult to interpret. I suggest either moving the entire Figure 2 to the Supplementary Materials or retaining only the two images that clearly depict vasodilation of the “feeding vessel.”

In Figure 3, there is a minor error: although vascular alteration is clearly demonstrated, vasoconstriction (Vc) is not labeled in Figure 3B.

Finally, I would like to comment on the concept of a “one gland with two acini” (see lines 152–154). Two salivary glands represent the standard anatomical arrangement in most Diptera, including mosquitoes, black flies, and biting midges. Similarly, sand flies also possess two salivary glands. The fact that these glands drain into a common salivary duct does not justify referring to them as a single gland. Mosquitoes likewise have a pair of salivary glands (left and right), each with its own lateral salivary duct. These ducts join to form a single common salivary duct, which then opens into the hypopharynx.

In summary, the correct anatomical arrangement in sand flies consists of paired glands, paired ducts, and a common salivary duct. I kindly ask the authors to adopt this terminology and revise the manuscript accordingly.

Reviewer #2: The study has provided unique insight into the blood feeding mechanics of sand flies. The authors develop innovative methods to analyse this and highlight that there are a number of unrecognised phases to pool feeding for these vectors which may have important implications for the delivery of saliva, it's action and the tramisssion of pathogens. It would have been good to have compared two main genera of Leishmania vectors (Lutzomyia and Phlebotomus) but I am satisfied that the results presented on Lu. longipalpis is sufficiently new to advance our knowledge.

Points to address:

Line 56: Phlebotomine sand flies are the only proven vector of Leishmania infantum - use of the word 'main' suggests that there are other vectors.

Line 63. Explain/qualify what vessel 'recruitment' is in the context of your results.

Line 86/87: Add a diagram here of the female sand fly head with labels of the different mouthparts to allow the reader to interpret the paper more easily.

Line 119: Add Peters et al. 2008 (Nathan C. Peters et al. ,In Vivo Imaging Reveals an Essential Role for Neutrophils in Leishmaniasis Transmitted by Sand Flies.Science321,970-974(2008).DOI:10.1126/science.1159194) and Giraud et al.2018 (Giraud E, Lestinova T, Derrick T, Martin O, Dillon RJ, Volf P, Műller I, Bates PA, Rogers ME. Leishmania proteophosphoglycans regurgitated from infected sand flies accelerate dermal wound repair and exacerbate leishmaniasis via insulin-like growth factor 1-dependent signalling. PLoS Pathog. 2018 Jan 19;14(1):e1006794. doi: 10.1371/journal.ppat.1006794. PMID: 29352310; PMCID: PMC5792026). - these paper identifiy the interaction of uninfected and infected sand fly bites with the wound healing processes of the host, which are exploited by Leishmania.

Line 177: Figure 1 - add dimensions to the customised feeding chamber.

Line 264: add "crop" in brackets after diverticulum to make it clearer.

Line 272: add this survival data to the supplementary information.

Line 279: Figure 4 - the acinus in panel F has released its saliva - please replace with a picture of one that remains intact, in which the intraluminal saliva can be observed (stained with bromocresol purple) - for comparison with panel E, stained with bromothymol blue.

Lines 425-428: Can you give some idea of how long the sandfly was engaged in these different phases of bloodfeeding?

Line 428: Can you explain what you mean by recruitment of vessels and if this has been observed for other vectors or in other contexts as I am unclear how this (physiologically) happens.

Line 432: Did you observe any sand flies to directly cannulate a vessel for feeding. If so, what proportion of flies do this and for how long?

Reviewer #3: Overall, while the study provides interesting and novel observations on the feeding behavior of Lutzomyia longipalpis using intravital microscopy. Several important aspects require clarification to strengthen the manuscript, in particular, key methodological details are missing, including sample sizes, experimental design, and the number of observed events, which limits reproducibility and interpretation of the results. Also, while the findings are insightful, certain limitations should be addressed in the discussion, such as the study was conducted in the absence of Leishmania parasites, as the presence of the parasite and its associated PSG is known to significantly modify sand fly feeding mechanics.

**********

PLOS authors have the option to publish the peer review history of their article (what does this mean?). If published, this will include your full peer review and any attached files.

If you choose “no”, your identity will remain anonymous but your review may still be made public.

**Do you want your identity to be public for this peer review?**  For information about this choice, including consent withdrawal, please see our Privacy Policy.

Reviewer #1: No

Reviewer #2: No

Reviewer #3: No

**Figure resubmission:**

While revising your submission, we strongly recommend that you use PLOS’s NAAS tool (https://ngplosjournals.pagemajik.ai/artanalysis) to test your figure files. NAAS can convert your figure files to the TIFF file type and meet basic requirements (such as print size, resolution), or provide you with a report on issues that do not meet our requirements and that NAAS cannot fix.

**Reproducibility:**

To enhance the reproducibility of your results, we recommend that authors of applicable studies deposit laboratory protocols in protocols.io, where a protocol can be assigned its own identifier (DOI) such that it can be cited independently in the future. Additionally, PLOS ONE offers an option to publish peer-reviewed clinical study protocols. Read more information on sharing protocols at https://plos.org/protocols?utm_medium=editorial-email&utm_source=authorletters&utm_campaign=protocols

# Author comment: Author response to Decision Letter 1

**DOI:** 10.1371/journal.pntd.0014067.r002

6 Aug 2026

Attachment Submitted filename: Response to Reviewers (1).docx

# Editor report: Decision Letter 1

**DOI:** 10.1371/journal.pntd.0014067.r003

**Authors:** Audrey Lenhart

10 Aug 2026

Dear Dr Aguiar Martins,

We are pleased to inform you that your manuscript 'New insights on the Feeding Site and Salivation by Lutzomyia longipalpis (Diptera: Psychodidae) During Blood Ingestion on Host Skin' has been provisionally accepted for publication in PLOS Neglected Tropical Diseases.

Before your manuscript can be formally accepted you will need to complete some formatting changes, which you will receive in a follow up email. A member of our team will be in touch with a set of requests.

Please note that your manuscript will not be scheduled for publication until you have made the required changes, so a swift response is appreciated.

IMPORTANT: The editorial review process is now complete. PLOS will only permit corrections to spelling, formatting or significant scientific errors from this point onwards. Requests for major changes, or any which affect the scientific understanding of your work, will cause delays to the publication date of your manuscript.

Should you, your institution's press office or the journal office choose to press release your paper, you will automatically be opted out of early publication. We ask that you notify us now if you or your institution is planning to press release the article. All press must be co-ordinated with PLOS.

Thank you again for supporting Open Access publishing; we are looking forward to publishing your work in PLOS Neglected Tropical Diseases.

Best regards,

Audrey Lenhart

Section Editor

PLOS Neglected Tropical Diseases

Audrey Lenhart

Section Editor

PLOS Neglected Tropical Diseases

Shaden Kamhawi

co-Editor-in-Chief

PLOS Neglected Tropical Diseases

orcid.org/0000-0003-4304-636XX

Paul Brindley

co-Editor-in-Chief

PLOS Neglected Tropical Diseases

orcid.org/0000-0003-1765-0002

***********************************************************

# Editor report: Acceptance letter

**DOI:** 10.1371/journal.pntd.0014067.r004

**Authors:** Audrey Lenhart

Dear Dr Aguiar Martins,

We are delighted to inform you that your manuscript, "New insights on the Feeding Site and Salivation by Lutzomyia longipalpis (Diptera: Psychodidae) During Blood Ingestion on Host Skin," has been formally accepted for publication in PLOS Neglected Tropical Diseases.

We have now passed your article onto the PLOS Production Department who will complete the rest of the publication process. All authors will receive a confirmation email upon publication.

The corresponding author will soon be receiving a typeset proof for review, to ensure errors have not been introduced during production. Please review the PDF proof of your manuscript carefully, as this is the last chance to correct any scientific or type-setting errors. Please note that major changes, or those which affect the scientific understanding of the work, will likely cause delays to the publication date of your manuscript. Note: Proofs for Front Matter articles (Editorial, Viewpoint, Symposium, Review, etc...) are generated on a different schedule and may not be made available as quickly.

Soon after your final files are uploaded, the early version of your manuscript will be published online unless you opted out of this process. The date of the early version will be your article's publication date. The final article will be published to the same URL, and all versions of the paper will be accessible to readers.

For Research Articles, you will receive an invoice from PLOS for your publication fee after your manuscript has reached the completed accept phase. If you receive an email requesting payment before acceptance or for any other service, this may be a phishing scheme. Learn how to identify phishing emails and protect your accounts at https://explore.plos.org/phishing.

Thank you again for supporting open-access publishing; we are looking forward to publishing your work in PLOS Neglected Tropical Diseases.

Best regards,

Shaden Kamhawi

co-Editor-in-Chief

PLOS Neglected Tropical Diseases

Paul Brindley

co-Editor-in-Chief

PLOS Neglected Tropical Diseases
